# Supplementary figures and images for: Comparative assessment of multiple COVID-19 serological technologies supports continued evaluation of point-of-care lateral flow assays in hospital and community healthcare settings
Source: PLoS Pathog. 2020 Sep 24;16(9):e1008817. doi: 10.1371/journal.ppat.1008817 (PMC7514033; doi:10.1371/journal.ppat.1008817)

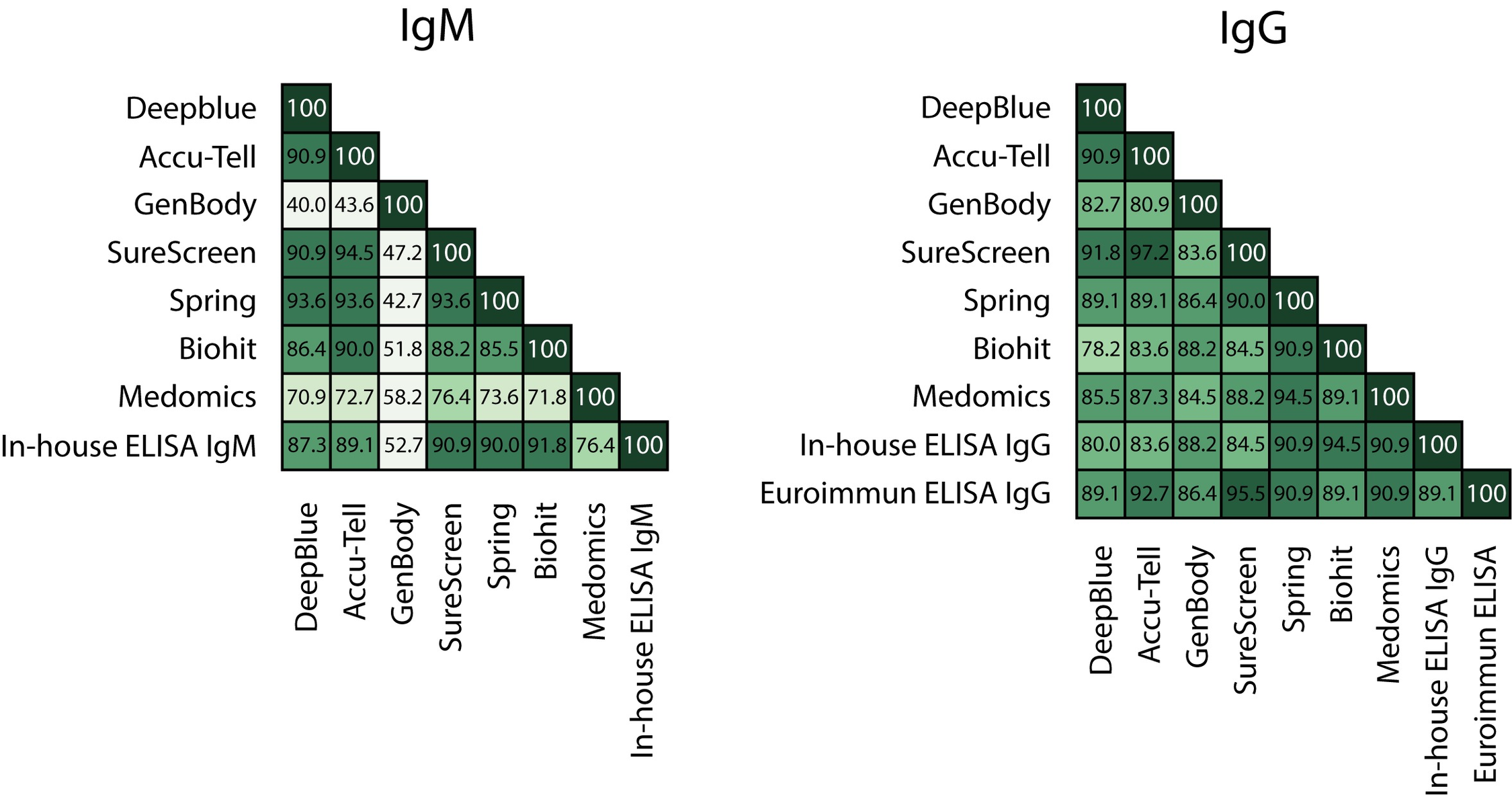

Supplement: S1 Fig — Serological assays were compared for IgM or IgG (left and right panels, respectively), and the percentage agreement between each of the samples in the assays is represented within each box. (TIF) [file ppat.1008817.s001.tif]

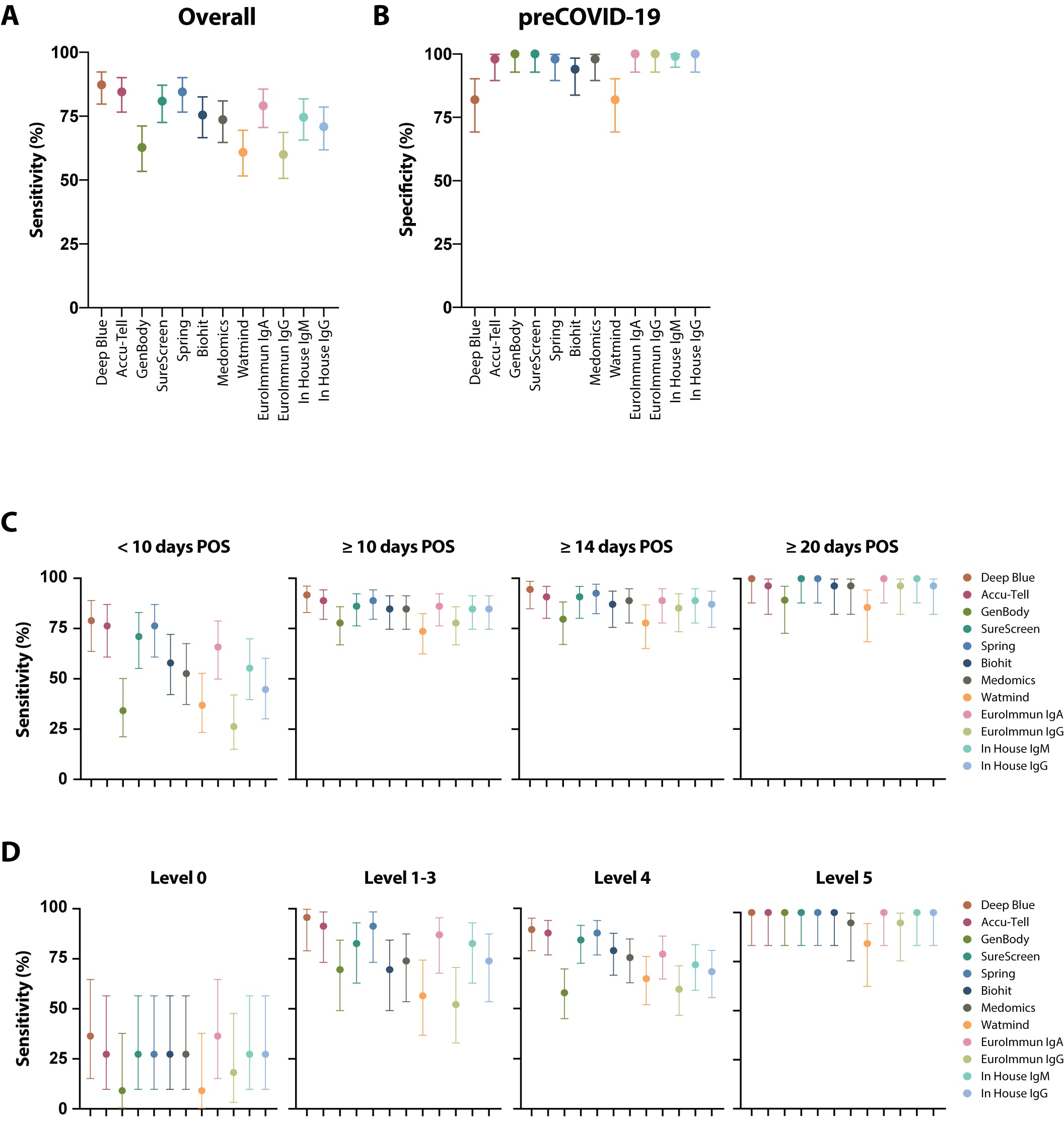

Supplement: S2 Fig — Overall sensitivity (A) and specificity (B) were determined for each serological assay as in Fig 5. Sensitivity was determined for each serological assay at increasing days POS (C), or severity of illness (D). Results for each test were either categorised according to whether the serum sample was from <10, ≥10, ≥14, or ≥20 days POS, or severity of illness, with 0 indicating mild illness (requiring no respiratory support) and 5 indicating critical (requiring ECMO) (see Materials and methods for full classification). 95% confidence intervals are shown for each assay in all panels (Wilson/Brown expected binomial). (TIF) [file ppat.1008817.s002.tif]
