# Supplementary material for: Comparative assessment of multiple COVID-19 serological technologies supports continued evaluation of point-of-care lateral flow assays in hospital and community healthcare settings
Source: PLoS Pathog. 2020 Sep 24;16(9):e1008817. doi: 10.1371/journal.ppat.1008817 (PMC7514033; doi:10.1371/journal.ppat.1008817)
Supplement: S1 Table — Specificity and sensitivity (%) were determined for each configuration of the in-house ELISA (detection of IgM and IgG to N, S and RBD) during initial development. 320 pre-pandemic samples from several cohorts were used as negative controls for specificity calculations, and 24 RT-PCR-confirmed SARS-CoV-2 positive samples were used as positive controls for sensitivity calculations. 95% CIs are shown for each calculation. (DOCX) [file ppat.1008817.s003.docx]

**S1 Table**

Sensitivity and specificity of in-house ELISAs during development phase, with 95% CI.
