# Supplementary material for: Comparative assessment of multiple COVID-19 serological technologies supports continued evaluation of point-of-care lateral flow assays in hospital and community healthcare settings
Source: PLoS Pathog. 2020 Sep 24;16(9):e1008817. doi: 10.1371/journal.ppat.1008817 (PMC7514033; doi:10.1371/journal.ppat.1008817)
Supplement: S2 Table — 50 pre-pandemic negative samples from the St Thomas’ emergency admissions cohort (STH Healthy, March 2019) were used to perform head-to-head specificity calculations for all immunoassay platforms. An extended panel of 105 samples was used for specificity calculations of the in-house ELISA for anti-S IgM and IgG. 95% CIs are shown for each calculation. (DOCX) [file ppat.1008817.s004.docx]

**S2 Table**

Head-to-head specificity calculations for all immunoassays
