# Supplementary material for: Comparative assessment of multiple COVID-19 serological technologies supports continued evaluation of point-of-care lateral flow assays in hospital and community healthcare settings
Source: PLoS Pathog. 2020 Sep 24;16(9):e1008817. doi: 10.1371/journal.ppat.1008817 (PMC7514033; doi:10.1371/journal.ppat.1008817)
Supplement: S3 Table — A select group of best-performing lateral flow immunoassays were taken forward for extended specificity calculations, using up to 200 pre-pandemic negative samples from the St Thomas’ emergency admissions cohort (STH Healthy, March 2019). 95% CIs are shown for each calculation. (DOCX) [file ppat.1008817.s005.docx]

**S3 Table**

Specificity of selected lateral flow immunoassays determined on an extended panel of pre-pandemic serum samples from March 2019
