# Supplementary material for: Comparative assessment of multiple COVID-19 serological technologies supports continued evaluation of point-of-care lateral flow assays in hospital and community healthcare settings
Source: PLoS Pathog. 2020 Sep 24;16(9):e1008817. doi: 10.1371/journal.ppat.1008817 (PMC7514033; doi:10.1371/journal.ppat.1008817)
Supplement: S4 Table — Head-to-head sensitivity calculations were performed for all immunoassays on a panel of 110 SARS-CoV-2-positive samples. Results for each test were further categorised according to whether the serum sample was from <10, ≥10, ≥14, or ≥20 days POS. 95% CIs are shown for each calculation. (DOCX) [file ppat.1008817.s006.docx]

**S4 Table**

Sensitivity of immunoassays classified by days POS
