# Supplementary material for: Comparative assessment of multiple COVID-19 serological technologies supports continued evaluation of point-of-care lateral flow assays in hospital and community healthcare settings
Source: PLoS Pathog. 2020 Sep 24;16(9):e1008817. doi: 10.1371/journal.ppat.1008817 (PMC7514033; doi:10.1371/journal.ppat.1008817)
Supplement: S5 Table — Head-to-head sensitivity calculations were performed for all immunoassays on a panel of 110 SARS-CoV-2-positive samples and classified according to severity of disease, with 0 indicating mild illness (requiring no respiratory support) and 5 indicating critical (requiring ECMO) (see Materials and methods for full classification). 95% CIs are shown for each calculation. (DOCX) [file ppat.1008817.s007.docx]

**S5 Table**

Sensitivity of immunoassays classified by disease severity
